# Application of Petrographic and Stereological Analyses to Describe the Pore Space of Rocks as a Standard for the Characterization of Pores in Slags and Ashes Generated after the Combustion of Municipal Waste

**DOI:** 10.3390/ma16247706

**Published:** 2023-12-18

**Authors:** Katarzyna Godyń, Barbara Dutka, Maciej Tram

**Affiliations:** Strata Mechanics Research Institute, Polish Academy of Sciences, Reymonta 27, 30-059 Kraków, Polandmaciej.tram@imgpan.pl (M.T.)

**Keywords:** microscopic analysis, petrography, porosity, slags and ashes, municipal waste

## Abstract

Slags and ashes generated in thermal waste treatment facilities require proper management. In line with the principles of the circular economy, new, more economical, and ecological possibilities for utilizing these substances are sought. These waste materials exhibit characteristics similar to rocks in many aspects. This study focuses on analyzing the similarities and differences between nine selected rocks and three samples of slags and ashes obtained from municipal waste incineration. The main research device used was a polarizing microscope, using reflected, transmitted, and fluorescent light. Additionally, low-pressure gas adsorption equipment, as well as helium and quasi-liquid pycnometers, were used for complementary analysis. The parameters analyzed mainly covered broadly defined petrographic properties of rocks and slags, with a particular emphasis on porosity, porous structure, and the spatial distribution of pores and fractures. The conducted analyses confirmed a significant similarity between slags and ashes and clastic sedimentary rocks such as sandstones and mudstones. The research results underscore the importance of petrographic microscopic studies for analyzing materials other than rocks. They also allow for exploring applications for slags and ashes in areas traditionally reserved for natural rock resources. The findings also indicate the necessity of using methods other than microscopic ones to describe the pore space of rocks. The lower measurement range of the optical microscope is limited to approximately 1 μm, covering only macropores. Other methods extend this characteristic to micro- and mesopores. Therefore, a combination of different methods is often employed to obtain a more comprehensive characterization of rock porosity.

## 1. Introduction

Among the diverse petrographic characteristics of rocks, issues related to porosity, porous structure, and the spatial distribution of pores and fractures deserve special attention. These parameters are essential factors influencing the physical properties of rocks such as their strength, deformability, and permeability [1], and are indispensable in many fields of science and industry. In the oil and gas industry, this data is required, among other things, for assessing the rocks’ capacity to accumulate crude oil or natural gas [2,3]. The porosity of rocks is a crucial parameter in geological and geophysical studies [4], aiding in determining the structural composition and properties of rocks [5] and describing the adsorption parameters of rocks [6,7,8,9]. Porosity analyses are instrumental in hydrogeological issues, such as the ability of groundwater to move in underground water reservoirs, water resource management, and source water protection [10,11]. Knowledge of porosity parameters also influences broadly understood environmental protection issues [12,13]. Porosity studies are also applied in geotechnical engineering in construction projects like roads, bridges, and buildings. Data on soil and rock porosity are essential for assessing soil bearing capacity and structural stability. Determining these properties is crucial for all design work where the bedrock is the basis for the constructed structures (road construction, residential, industrial, etc.) or is the medium in which these activities are subsequently carried out (tunnel construction, underground mining, creation of underground gas and liquid tanks, geological waste repositories, etc.) [14,15,16,17,18].

Characteristic for rocks, the large diversity in the shape, size, and distribution of pores implies a series of metrological challenges in determining the parameters describing the rock structure. The pore space can be characterized by examining individual petrophysical parameters. According to [19], porosity is a petrophysical quantity that can be defined as the ratio of the volume of empty space to the total volume of the analyzed rock (or other porous material). To understand the complexity of this issue, it should be noted that porosity varies depending on the type of rock. Sedimentary rocks have different porosity parameters than igneous or metamorphic rocks. In sedimentary rocks, porosity changes at various stages of the rock-forming processes, from sedimentation through diagenesis to epi- and diagenetic processes [20,21]. Porosity associated with sedimentation processes is called primary or syngenetic porosity. As a result of processes occurring during diagenesis, secondary (epigenetic) porosity is formed [22,23]. Primary porosity is primarily intergranular porosity. Secondary porosity is particularly developed in carbonate rocks, leading to the filling of primary pores and/or recrystallization of secondary minerals [21,24,25,26].

In igneous rocks, the structure of the pore space depends on the type and chemical composition of the magma, as well as the conditions of its crystallization [21]. Plutonic igneous rocks usually have a massive structure, practically without the presence of intercrystalline pores. In these rocks, porosity is almost exclusively associated with fractures formed during or after the crystallization process of minerals from the magma. In volcanic igneous rocks, sometimes the crystallization conditions and magma composition favor the occurrence of pores that form during the rapid cooling of gas-rich magma [27]. Some volcanic rocks may have numerous, sometimes very large, often oval-shaped pores. Extremely large porosities are found, for example, in various types of pumices [28].

Rocks contain pores of various sizes and shapes, which significantly influence the permeability of the rock for fluids and gases, as well as determining many other rock properties. From the perspective of pore connectivity, open pores, which are interconnected and form open porosity, and closed pores, which are connected and form total porosity, are distinguished [29]. Based on this classification, in practice, total and effective porosity coefficients are distinguished, which characterize the capacity and storage capabilities (e.g., hydrocarbons) of the analyzed rocks.

To determine the sizes and shapes of pores, classifications based on various criteria are introduced. From the perspective of pore shapes, they can be divided into: stereopores (voids whose sizes in three mutually perpendicular directions are approximately equal), channels (channel-like pores, where the size in one direction is significantly larger than the sizes in the other two directions), and fractures (size in one direction is orders of magnitude smaller than the sizes in the other two directions) [24,30,31,32]. According to [33], porous materials are divided into three groups: microporous—pore diameter < 2 nm; mesoporous—pore diameter from 2 to 50 nm; macroporous—pore diameter > 50 nm.

Basic petrographic analyses of rocks are usually conducted using polarizing microscopes [8,25,34,35]. To expand the methodology, X-ray analyses, SEM, EDS, etc., are also employed. However, when characterizing the porosity of rocks, there are several research methods available. The choice of an appropriate method depends on the type of rock, its properties, and the research objectives. In practice, a combination of different methods is often used to obtain a more comprehensive characterization of rock porosity. Porosity can be determined through laboratory testing or geophysical methods [36,37]. Laboratory research methods include porosimetry techniques, such as mercury porosimetry and helium porosimetry. Mercury porosimetry is used for measuring porosity, especially in rocks with low permeability and high porosity. Helium porosimetry serves as an alternative to mercury porosimetry, as it is more environmentally friendly and safer for operators. Its application depends on specific research goals and the type of rocks under investigation [29,38,39].

Another group of methods characterizing pore space includes microscopic imaging techniques. These methods utilize optical microscopy, electron microscopy, and micro-computed tomography (micro-CT). These techniques allow for obtaining images of the pore space in rocks and assessing their porosity and pore structure [40,41,42]. However, these methods also have limitations and may not always accurately represent the distribution and spatial arrangement of pores. Optical microscopy and micro-CT can provide excellent results, mainly for macropores. On the other hand, scanning electron microscopy allows for studying the geometry and shape of pores but may face challenges in quantitative pore analysis.

There are also other methods that support the study of the pore space of materials and, depending on the pore size range, contribute to the overall assessment of the internal structure of various types of materials. Methods for assessing texture parameters include low-pressure adsorption methods based on the analysis of adsorption/desorption isotherms as well as densimetric methods enabling the assessment of the total and effective porosity [43,44].

Waste generated as a result of human activities is an inherent element of the modern world and poses a significant problem in the economy of every country. According to data from the Central Statistical Office in Poland, in 2022, 13.4 million tons of municipal waste and 115 million tons of industrial waste were collected [45]. In Poland, waste disposal through landfill is one of the primary methods of waste removal [46]. Consequently, there are numerous municipal and industrial waste disposal sites marked on the country’s map. However, there is a current effort to reduce landfilling and promote methods such as processing, recovery, and recycling of waste. Companies and institutions dealing with waste are making extensive efforts to utilize the generated waste for various practical applications and reintroduce these materials as products into a closed-loop economy. Proper waste management aims to minimize the negative environmental impacts, including water, air, and soil pollution, as well as to promote the reduction in greenhouse gas emissions. Appropriate waste management is crucial for environmental protection and the preservation of natural resources for future generations. The main challenge lies in two types of waste: municipal waste, generated by an average citizen, and industrial waste [47,48,49]. In recent decades, social awareness, especially in Europe, has noticeably increased, leading to a higher degree of waste segregation. Various facilities, including those utilizing calorific waste for alternative fuel production, are being established. Different methods of waste utilization are being explored, such as incorporating waste into the production of concrete, e.g., [50,51]. However, a significant amount of municipal and industrial waste still ends up in landfills. One method to reduce the quantity of generated waste and mitigate its impact is incineration in specially designated waste-to-energy plants [47,48]. The primary by-products of incineration in such facilities are “furnace slag and ash” [52,53]. These are solid wastes, predominantly composed of the sand and gravel fraction, with silt accounting for only a small percentage [44]. During incineration, waste is reduced by approximately ¾ (in mass) [46,47]. While waste-to-energy plants mitigate the majority of the original waste mass, the remaining ¼ in the form of slags and ashes still requires proper management. Hence, there is a need to look for effective methods of managing these waste materials, to reintroduce them as a product into the circular economy [54].

The authors of the paper, while testing slags and ashes resulting from municipal waste incineration, observed that these materials had many rock-like characteristics.

The aim of the study was to identify common points between the rock and waste, examine the characteristics of different types of rocks, relate them to waste materials, and determine which rocks are most similar to the analyzed slag–ash materials in terms of petrographic features. The innovative nature of the work will enable a more effective exploration of the applications for waste materials, facilitating their integration into a circular economy.

It is highly probable that materials resulting from waste incineration are or will be used in the economy similarly to rock materials (e.g., in various types of construction work, road foundations, hydraulic engineering structures, etc.).

## 2. Materials and Methods

### 2.1. Samples

**Figure 1:**
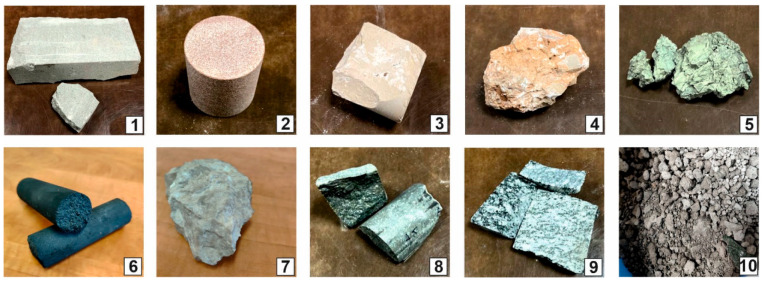
Samples of raw rocks and slag analyzed in the study. 1—quartz sandstone PRO1, 2—quartz sandstone PRO2, 3—micritic limestone PRO3; 4—carbonate breccia PRO4; 5—marl shale PRO5; 6—graphite PRO6; 7—organodetritic limestone PRO7; 8—basalt PRO8; 9—granite PRO9; 10—post-process slag and ash PRO10.

To achieve the goal of the study, nine rocks were selected, characterized by different composition, structure, and genesis. They originate from Polish rock formations of various ages and include: two quartz sandstones (PRO1 and PRO2), micritic limestone (PRO3), carbonate breccia (PRO4), marl shale (PRO5), graphite (PRO6), organodetritic limestone (PRO7), basalt (PRO8), granite (PRO9), and a material other than rock, namely post-process slag (PRO10), generated from municipal waste incineration (Figure 1). The materials for research were obtained through collaboration with one of the waste incineration plants operating in Poland. It should be noted that the analyzed graphite, although composed of natural materials, is a product of anthropogenic origin, but with properties similar to coal. It is a product created for the production of graphite electrodes according to [55]. “Graphite electrodes are large cylindrical structures consisting mainly of petroleum coke, coniferous coke and coal bitumen, which is used as a cement. They are produced by calcination, mixing, kneading, pressing, roasting, graphitization, and mechanical processing”.

### 2.2. Microscopic Analysis

**Figure 2:**
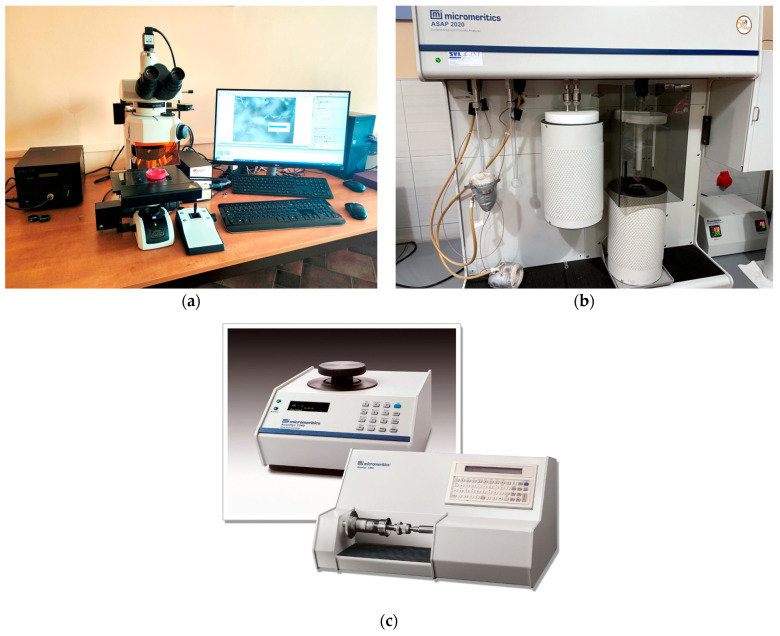
Laboratory devices used for analyses. (**a**)—Nikon LV100POL polarizing microscope, Prior Lumen200 fiber optic fluorescence, and Nikon NIS Elements software; (**b**)—ASAP 2020 advanced specific surface area and porosity analyzer; (**c**)—Micromeritics pycnometers: helium AccuPyc 1340 (**left**) and quasi-fluid GeoPyc 1360 (**right**).

With the selected rocks, petrographic–stereological analyses were conducted, with particular emphasis on describing the pore space (its size and characteristic parameters were determined). Subsequently, similar analyses were performed on three samples of post-process slag collected from municipal waste incineration plants. Microscopic analyses were conducted on all three samples, and it was decided that, due to small differences in the properties of the slags, the mixed material would be used for further analysis. Various types of measuring devices were employed to determine the petrographic and structural–textural parameters in different ways and at different magnification levels. The devices used for the analyses include: a Nikon LV100POL polarizing microscope with Marzhauser *X*, *Y*, and *Z*-axis motorization, Prior Lumen200 fiber optic fluorescence system, and Nikon NIS Elements image analysis software (Figure 2a).

The observation was conducted at a wavelength ranging from 320 to 400 nm. Petrographic studies were carried out in both transmitted light (thin sections) and reflected light (polished section). Stereological analyses were also performed using quantitative point analysis. The pores and fractures present in each rock and waste sample were counted. It is important to note that the results of these analyses allowed estimating the proportion of large pores in the examined structure, focusing only on the voids visible under the optical microscope magnification (i.e., above 0.001 mm in size). Stereological analyses were conducted on thin sections and polished mounts based on the Cavalieri–Hacquet principle, which states that the “percentage content of a given phase in the volume of an alloy (in this case, rock) on the plane of the section is the same as the length of the straight line” [56].

### 2.3. Adsorption Measurements

The texture of the materials was analyzed with the low-pressure adsorption method using the ASAP 2020 analyzer (Micromeritics) (Figure 2b). For this purpose, the adsorption and desorption of gas (N_2_) were measured at a liquid nitrogen temperature of −196 °C. Adsorption points were determined in the range of relative pressure 0 < p/p° < 0.99, determined as the ratio of the measured pressure p to the saturation vapor pressure p° of pure nitrogen at the measurement temperature. Samples with a grain fraction of 0.16–0.25 mm and a mass of approximately 1 g were dried at 105 °C. The appropriate amount of the tested sample was selected based on the expected adsorption capacity of the material. First, in the preparation port of the ASAP2020 analyzer, the samples were subjected to heating at 80° and degassing for the 12 h procedure. Then, the prepared samples were placed in the measuring system of the analyzer and the measurement was performed. The amounts of adsorbed or desorbed nitrogen per unit mass of the sample (cm^3^STP/g) were measured depending on the equilibrium relative pressure p/p°. Nitrogen adsorption in the full range of relative pressures enabled the characterization of the internal structure (texture) of the material in the range from microporosity to mesoporosity, i.e., p/p° values close to 1, where the capillary condensation occurs and the adsorbate turns into a liquid state.

Based on the measured nitrogen adsorption–desorption isotherms at −196 °C, the BET adsorption (Brunauer–Emmett–Teller) and the BJH (Barrett–Joyner–Halenda) models were calculated. The BET method is a procedure for determining the specific surface area of the samples obtained from the linear form of the equation in the range of relative pressures 0.05 < p/p° < 0.30:

(1) $$\frac{p/p^{\circ }}{a\left(1-p/p^{\circ }\right)}=\frac{1}{a_{m}C}+\frac{\left(C-1\right)}{a_{m}C}p/p^{\circ }\;$$

where: a (mol/g) is the equilibrium adsorption, am (mol/g) is the capacity of the adsorption monolayer, C (−) is the equilibrium constant, p/p° (−) is the relative pressure, p (bar) is the absolute pressure, p (bar) is the saturated vapor pressure of the adsorbate.

From the parameters of the line fitted to the adsorption data using the least squares method, the monolayer capacity am and the constant C were determined. Assuming that the material surface was covered with a monolayer consisting of adsorbed gas molecules, the specific surface area S*_BET_* was determined from the following equation:

(2) $$S_{BET}=a_{m}\omega N_{A}\;\left[\frac{m^{2}}{g}\right],$$

where: ω (nm^2^) is the seating surface—area occupied by the molecule of adsorbate at the interface, N_A_ (mol^−1^) is the Avogadro constant.

Thus, the outer surface and inner surface of the pores, which constituted the total specific surface area of the material, were assessed.

The Barrett–Joyner–Halenda (BJH) model was used to determine the distribution and size of the pores, including the occupied volume and pore area. The method made it possible to determine the distribution of pore sizes in the range of micropores and mesopores. The average pore diameter (4 V/A) was also determined. Faass correction was applied [57]. The BJH method enabled the analysis of the surface properties of samples based on the assumption of capillary condensation. A graphical pore size distribution was obtained, along with incremental and cumulative values. Assessment of the shape of a possible hysteresis loop allowed for the determination of the pore geometry.

### 2.4. Densimetric Research

An important stage of the study was the assessment of the pore space of the materials, based on the measurements of real and apparent density. The real density was determined by helium pycnometry and the apparent density was determined by quasi-liquid pycnometry using the AccuPyc 1340 and GeoPyc 1360 (Micromeritics) analyzers, respectively (Figure 2c). The helium pycnometer precisely determined the real density, which was the ratio of the skeleton mass to its total volume. A quasi-liquid pycnometer measured the apparent density of materials by taking into account the apparent volume (including pores). Based on both densities, the porosity of the samples and the specific pore volume were determined as follows:

(3) $$\varepsilon =\left(1-\frac{\rho_{p}}{\rho_{r}}\right)$$

(4) $$V_{p}=\frac{1}{\rho_{p}}-\frac{1}{\rho_{r}},$$

where:

ε—porosity, -,

$V_{p}$—specific pore volume, cm^3^/g,

$\rho_{r}—$real density, g/cm^3^,

$\rho_{p}$—apparent density, g/cm^3^.

## 3. Results

### 3.1. Microscopic Analyses

**Figure 3:**
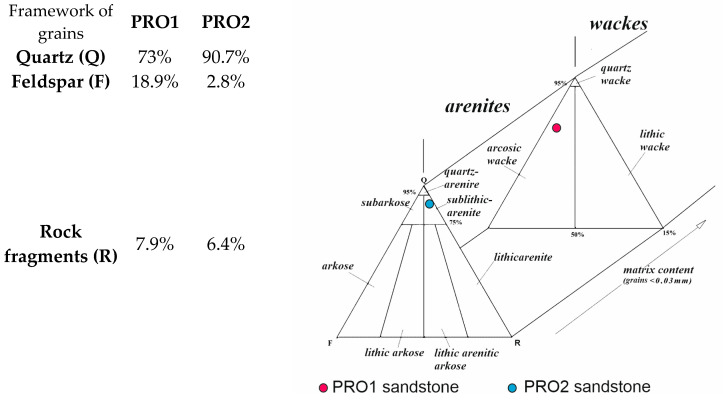
Composition of the grain skeleton of sandstones along with the classification triangle according to [58].

**Figure 4:**
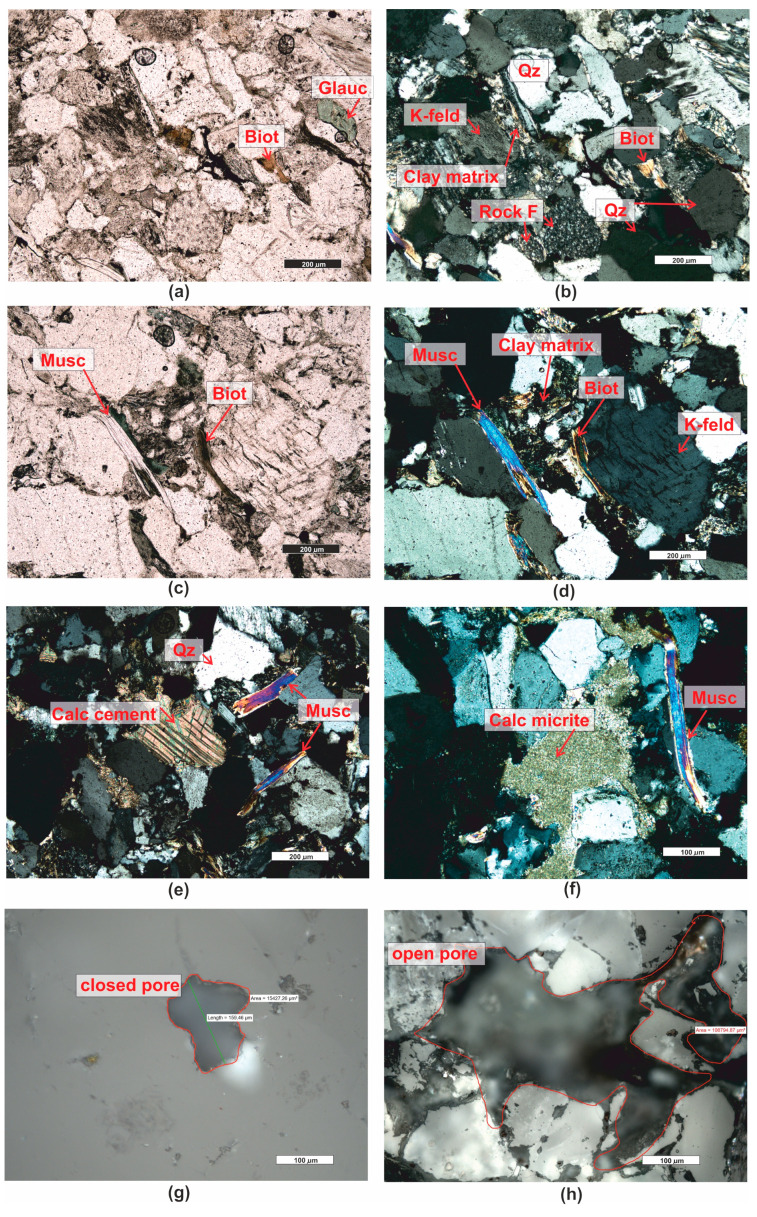
Microscopic photographs of sandstone PRO1. (**a**) transparent light, thin section, PPL; (**b**) transparent light, thin section, XPL; (**c**) transparent light, thin section, PPL; (**d**) transparent light, thin section, XPL; (**e**) transparent light, thin section, XPL; (**f**) transparent light, thin section, XPL; (**g**) reflected light, polished section, PPL; (**h**) reflected light, polished section, PPL. Legend: Biot—biotite; Glauc—glauconite; Musc—muscovite; Qz—quartz; Feld—feldspar; K-feld—potassium feldspar, Plag—plagioclase; Calc—calcite; Rock F—rock fragments; Calc micrite—calcitic micrite, Calc sparite—sparry calcite cement, Car—carbonates, Ol—olivine, Idd—iddinksite, org—organogenic remains.

*Sandstone (PRO1)* is a rock characterized by a light gray color (Figure 1), a psammitic structure, a fine-grained, and random texture. The dominant components of the grain framework are quartz grains of varied sizes, comprising as much as 73% by volume according to quantitative analysis (Figure 3). In larger grains of quartz, undulatory extinction is visible. The grains exhibit a low degree of rounding and are typically sub- and anhedral (Figure 4b,c,e,f). The rock also contains potassium feldspar and plagioclase (Figure 4b,d), which usually show an advanced degree of weathering. Their percentage content is 18,9% (Figure 3). Another component building the sandstone’s framework is rock fragments, usually well-rounded, anhedral pieces mainly composed of quartzites (Figure 4b,e). They constitute 7.9% of the rock (Figure 3). The presence of micas is observed in small amounts in the rock, represented by muscovite and biotite. Muscovite forms well-preserved, elongated shapes, often contorted at the contact with other grains (Figure 4c–f). Biotite flakes, exhibiting a brown pleochroism, occur in minimal amounts and are strongly altered specimens (Figure 4a–d). The rock’s porous type cement consists of clay minerals (Figure 4b,d), sparitic and micritic calcite (Figure 4e,f), and glauconite (Figure 4a). The clay matrix (Figure 4b,d) is present in approximately 19% of the rock, while the cement, mainly carbonate (Figure 4e), is observed at around 7%.

In accordance with the classification by [58], the rock was categorized as arkosic wacke (Figure 3).

The rock’s porosity falls into the category of intergranular and intragranular types, primarily open porosity. The total porosity determined on polished sections is approximately 17.5% by volume. Sample pores occurring in the rock are illustrated in the photographs (Figure 4g,h).

**Figure 5:**
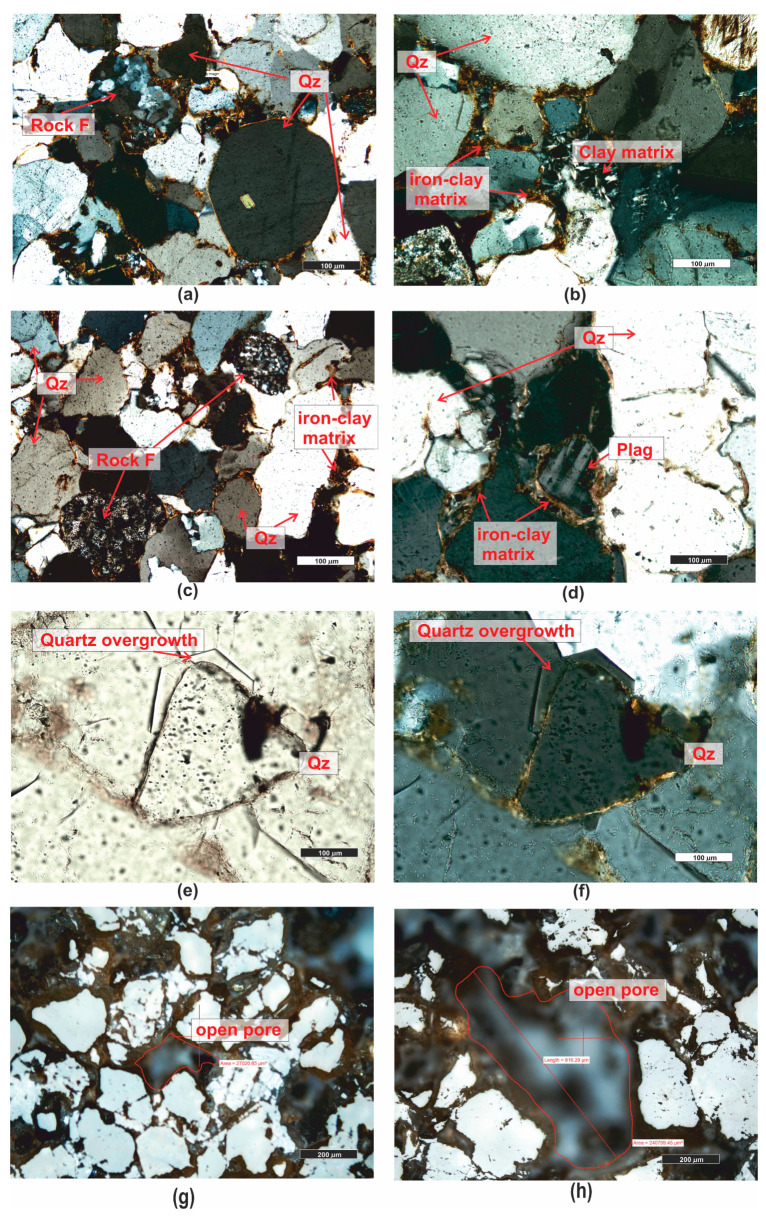
Microscopic photographs of sandstone PRO2. (**a**) transparent light, thin section, XPL; (**b**) transparent light, thin section, XPL; (**c**) transparent light, thin section, XPL; (**d**) transparent light, thin section, XPL; (**e**) transparent light, thin section, PPL; (**f**) transparent light, thin section, XPL; (**g**) reflected light, polished section, PPL; (**h**) reflected light, polished section, PPL. Legend: see Figure 4.

The cement of the sandstone is siliceous-iron-clayey, of the contact-porous type. The siliceous cement occurs mainly in the form of regenerative rims (Figure 5e,f) surrounding detrital quartz grains, occasionally forming silica clusters in the pore space. Iron compounds and clay minerals create coatings on the grains (Figure 5b–d) and are also present as irregular clusters in the pore space (Figure 5b).

*Sandstone* (*PRO2*) is a rock with a reddish hue (Figure 1), having a psammitic structure, a medium-grained, and random texture. The main sandstone’s grain framework are well-rounded, anhedral grains of quartz (Figure 5a–d), and partially sub- and euhedral (Figure 5e,f) with diameters ranging from 0.1 to 1.0 mm. The quartz grains are often surrounded by an iron substance (hence the reddish sediment color). These grains are moderately well-sorted. Quantitative analysis revealed that quartz constitutes almost 91% of the volume of the grain framework (Figure 3). The remaining components of the framework consist of rock fragments (Figure 5a,e), rounded, mainly siliceous, constituting approximately 6.5% of the rock’s framework (Figure 3), and feldspar grains, primarily plagioclase (Figure 5d). They represent only 2.8% of the volume in the framework (Figure 3). Additionally, single grains of glauconite, biotite, and muscovite were observed in the rock.

According to [58], the analyzed rock is sublithic arenite (Figure 3).

Point analysis revealed that the rock has a porosity of about 11.9%, and it is an open porosity. The pores in the rock are almost exclusively intergranular (Figure 5g,h), and there is also secondary infill of the pore space, observable in the form of siliceous regenerative rims (Figure 5e,f).

**Figure 6:**
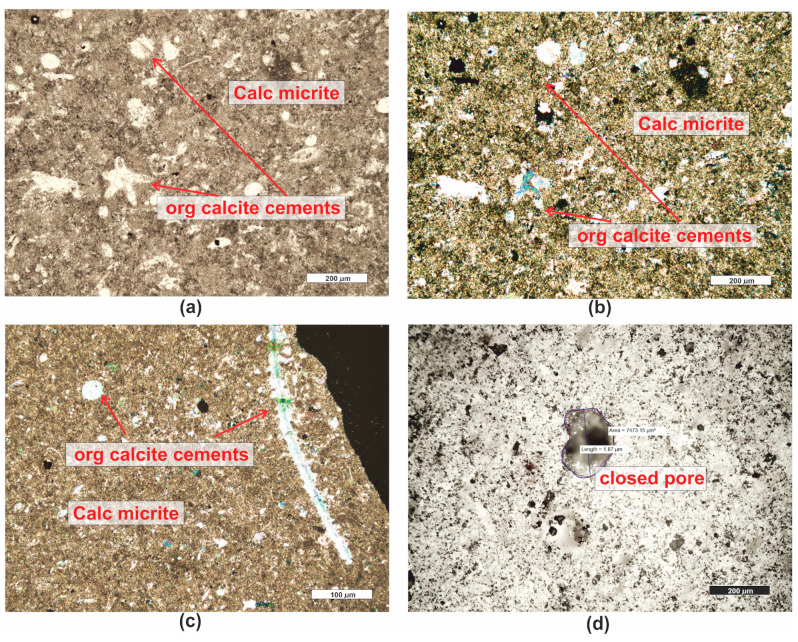
Microscopic photographs of limestone PRO3. (**a**) transparent light, thin section, PPL; (**b**) transparent light, thin section, XPL; (**c**) transparent light, thin section, XPL; (**d**) reflected light, polished section, PPL. Legend: see Figure 4.

*Micritic limestone (PRO3)* is a rock with a cream–gray color (Figure 1). It has a random texture, sometimes slightly porous, and a micritic structure. Microscopic examination reveals a micritic rock matrix (Figure 6a–c), in which fine fragments of sparitic calcite crystals (Figure 6a–c) and organogenic remains of varying sizes are embedded (Figure 6a–c). The rock is almost monomineralic, consisting of micritic and sparitic calcite. Occasionally, small, opaque ore minerals are visible. According to [59], the rock is classified as wackestone.

Numerous, although small, pores were observed in the rock (Figure 6d). These pores have irregular shapes and a complex surface, usually being intergranular. The total porosity of this material, determined microscopically, is approximately 11.6%.

**Figure 7:**
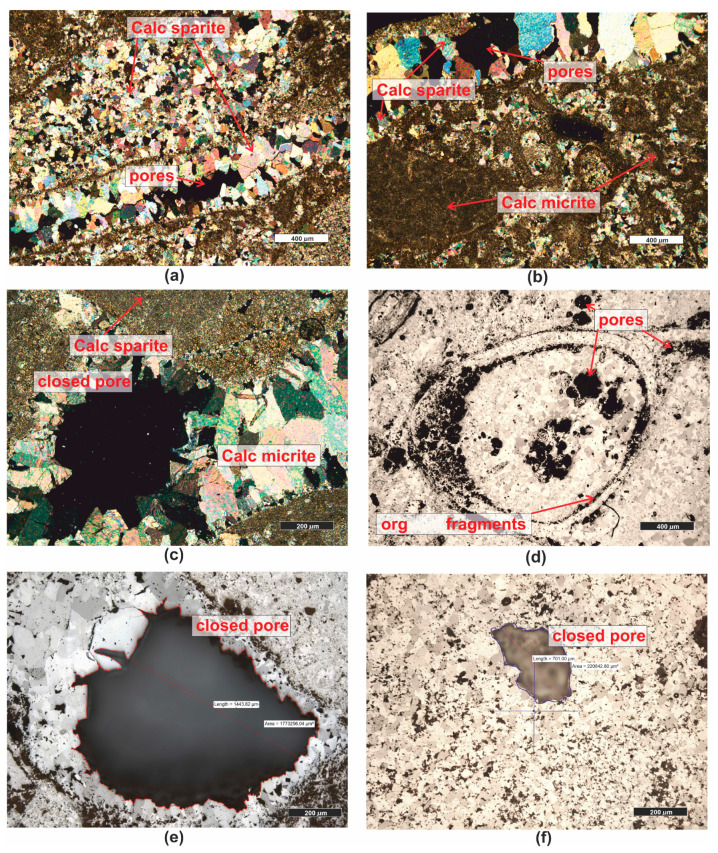
Microscopic photographs of limestone PRO4. (**a**) transparent light, thin section, XPL; (**b**) transparent light, thin section, XPL; (**c**) transparent light, thin section, XPL; (**d**) reflected light, polished section, PPL; (**e**) reflected light, polished section, PPL; (**f**) reflected light, polished section, PPL. Legend: see Figure 4.

*Carbonate breccia (PRO4)* is a rock with a reddish-cream color (Figure 1). It consists of lithified carbonate fragments, between which a reddish fine sediment of the “terra rossa” type is visible (this is a reddish sediment filling karst cavities, formed as a result of limestone karstification in a warm climate). It consists of hydroxides, hydrated aluminum oxides, and iron hydroxide [60]. The texture of the rock is random, slightly porous, and it crumbles easily into smaller fragments. Two types of sediment are visible in the microscopic image—these are fragments of micritic calcite (Figure 7a–c) and sparitic (Figure 7a–c) and microsparitic calcitic, or dolomitic fragments. Between the carbonates, reddish-brown terra-rossa-type sediments are visible. The rock has numerous pores, mainly of the intergranular type (Figure 7a–c,e,f) but also intragranular (Figure 7d). The pores are primarily located between the crystals of dolomite. Such sediments are formed through epigenesis, resulting from the dolomitization of limestones under the influence of circulating solutions rich in Mg and CO_2_. The transformation of limestone sediment into dolomite involves a volume reduction of up to 12.3%, which is why numerous secondary dolomites are porous and cavernous [21]. These pores have various sizes, ranging from the size of individual μm^2^ to about 2 mm^2^ (Figure 7e,f). These pores are surrounded by sharp edges of crystals. The total porosity of this sediment, determined microscopically, is approximately 10.4%.

The rock, due to being a conglomerate of different carbonate fragments, exhibits characteristics of both mudstone and grainstone, as well as sparitic limestones [59].

**Figure 8:**
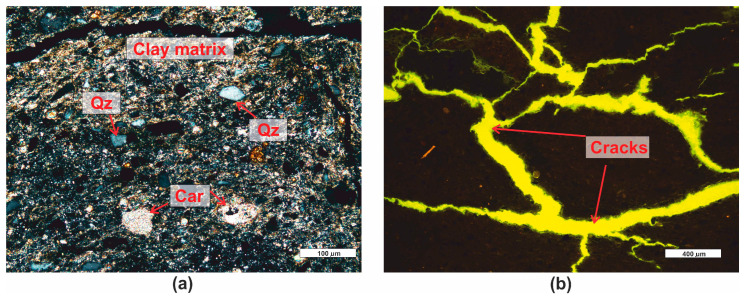
Microscopic photographs of marl shale PRO5. (**a**) transparent light, thin section, XPL; (**b**) fluorescent light (λ = 320–400 nm), polished section. Legend: see Figure 4.

*Marl shale (PRO5)* is a soft and crumbly dark-gray-colored sediment with a pelitic structure and a slightly parallel texture. This sediment contains clay minerals, densely distributed throughout the rock, among which there are fine microsparitic carbonates (Figure 8a). Occasionally, clusters of carbonates form oval enclaves. Among the clay minerals and carbonates, numerous grains of detrital quartz are present. Quartz occurs in the form of poorly rounded, subhedral grains (Figure 8a). The total pore space of the rock is impossible to determine and identify at microscopic magnifications. Clayey rocks have pores in the range of microporosity [32], which are invisible at the available magnifications in optical microscopes. The rock also contains macropores in the form of numerous fractures that align directionally in some places. These fractures are easily identifiable using fluorescence (Figure 8b). The porosity determined microscopically (macroporosity) is approximately 3%.

**Figure 9:**
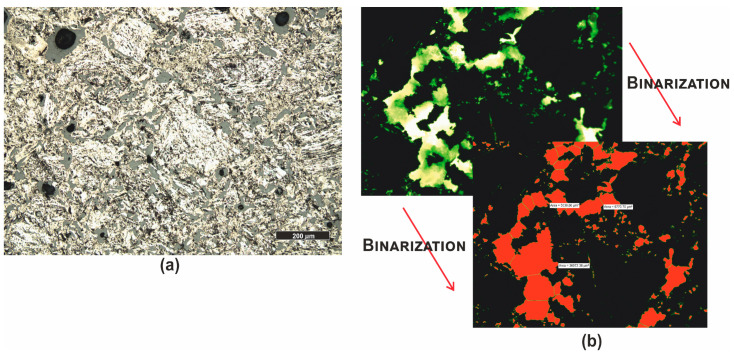
Microscopic photographs of graphite PRO6. (**a**) reflected light, polished section, PPL; (**b**) fluorescent light (λ = 320–400 nm), polished section and binarization.

*Graphite (PRO6)* is black in color (Figure 1) and has a compact and non-dispersible form. Microscopic analysis revealed significant similarities between the analyzed sample and certain cokes [61]. The sample image is shown in the photograph (Figure 9a), and due to the very high porosity of the sample, pore counting (total porosity) was performed using automatic image analysis. A series of microscopic images were taken in fluorescent light, then, in the Nikon Nis Elements program, the images were subjected to binary analysis (Figure 9b), and the total porosity of the sample was determined to be around 25%.

**Figure 10:**
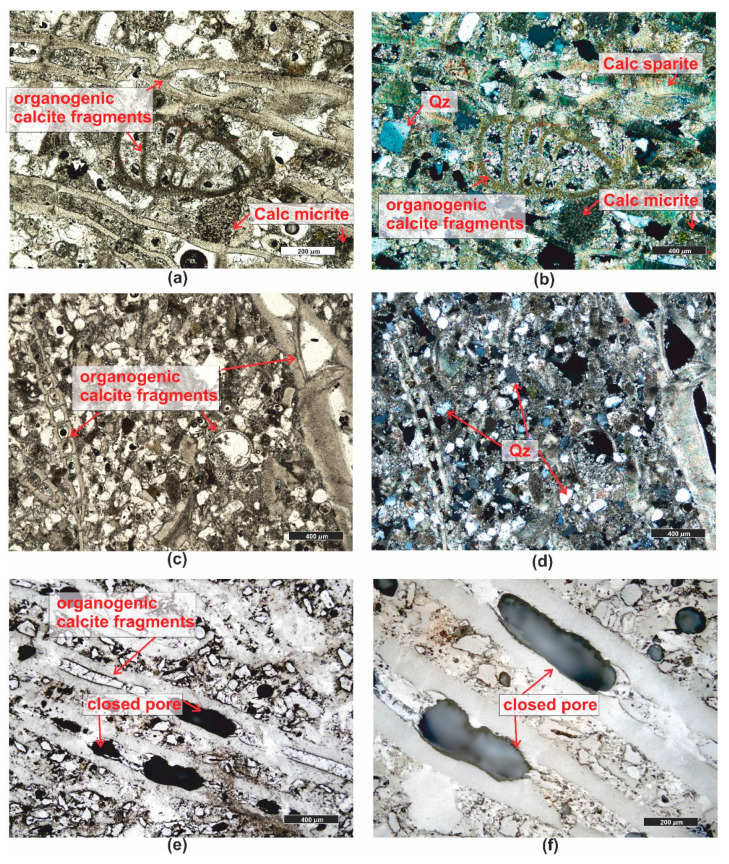
Microscopic photographs of organogenic limestone PRO7. (**a**) transparent light, thin section, PPL; (**b**) transparent light, thin section, XPL; (**c**) transparent light, thin section, PPL; (**d**) reflected light, polished section, XPL; (**e**) reflected light, polished section, PPL; (**f**) reflected light, polished section, PPL. Legend: see Figure 4.

*Organogenic limestone (PRO7)* has a cream–gray color with a micritic and random, porous texture (Figure 1). In the microscopic image, granular components are visible, usually micritized, such as intraclasts and pellets. The largest group of grain components constituting the rock’s framework consists of various organic remnants (bioclasts) (Figure 10a–f), including shells, snails, sponges, etc. Granular rock components are sometimes cemented by microsparitic crystals of carbonates. The rock has a significant admixture of detrital quartz grains which are poorly rounded (Figure 10b,d).

Due to the high content of mainly calcareous organic remnants, the rock is classified as organodetrital limestone of the grainstone type [59].

The limestone is highly porous, and it is dominated by moldic porosity, i.e., intragranular. In the grains, typically bioclasts, recrystallization or dissolution occurs, creating voids of large dimensions (Figure 10e,f). The total porosity of the investigated rock is as high as 26.4%.

**Figure 11:**
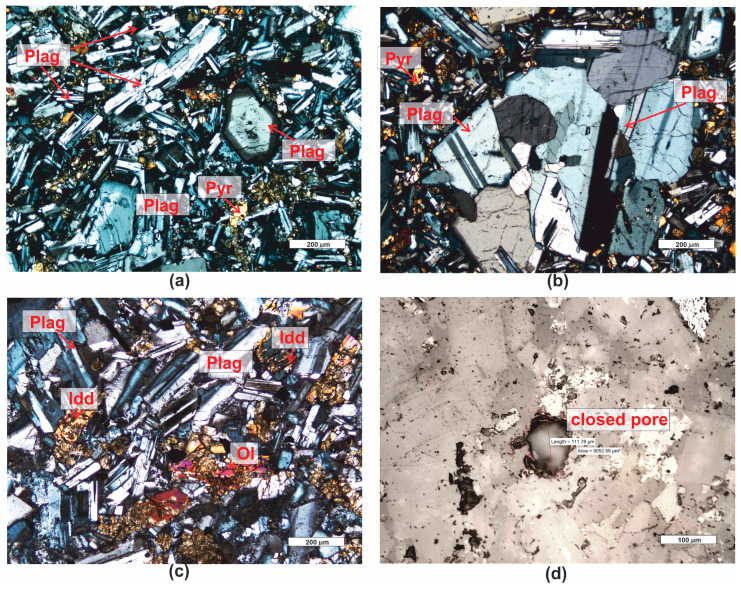
Microscopic photographs of basalt PRO8. (**a**) transparent light, thin section, XPL; (**b**) transparent light, thin section, XPL; (**c**) transparent light, thin section, XPL; (**d**) reflected light, polished section, PPL. Legend: see Figure 4.

*Basalt (PRO8)* belongs to the group of igneous volcanic rocks. It has a holocrystalline, inequigranular, porphyritic structure. The groundmass is composed of tabular, small euhedral crystals of plagioclase (Figure 11a–c), typically multiple twinned, and irregular, altered fragments of olivine, partially serpentinized, and pyroxenes (Figure 11a–c). These crystals are highly weathered. The groundmass is developed in the form of an ophitic structure (occurring in a rock consisting of elongated crystals of plagioclase with varying orientations, with interstitial xenomorphic grains of pyroxene and olivine filling the space between them). Phenocrysts embedded in the ophitic groundmass are repeatedly twinned plagioclases, sometimes exhibiting a zonal structure of crystals (Figure 11a).

Among the crystals, there are pores resulting from the specific crystallization of magmatic rocks—extrusive rocks, in which, due to the rapid cooling of magma, gas bubbles migrate, creating voids—pores (Figure 11d). The microscopic porosity of the rock is approximately 1.5%.

**Figure 12:**
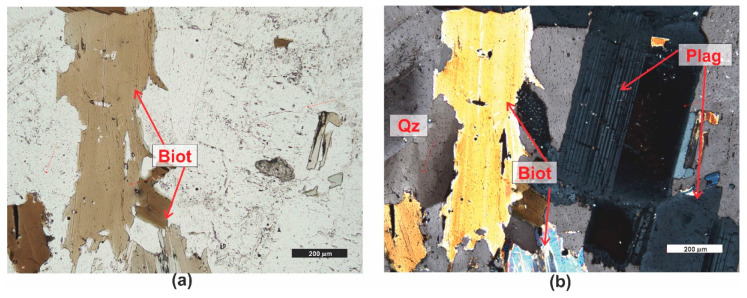
Microscopic photographs of granite PRO8. (**a**) transparent light, thin section, PPL; (**b**) transparent light, thin section, XPL. Legend: see Figure 4.

*Granite (PRO9)* is a gray (Figure 1) plutonic magmatic rock characterized by a holocrystalline, medium to fine crystalline, inequigranular structure. It has a compact, random texture, with only the micas aligning slightly in a directional manner. The minerals present in granite include quartz, with crystals having an irregular shape (Figure 12b), and potassium feldspar, occurring as large irregular or tabular crystals. On the surface of the crystals, there are dull areas resulting from weathering processes (Figure 12a,b), and plagioclases that occur as repeatedly twinned crystals (Figure 12b). The shape of the crystals is usually subhedral, tabular. Plagioclases are occasionally slightly cracked and weathered. Biotite, which appears abundantly in the rock (Figure 12a,b), is slightly oriented and exhibits brown or greenish pleochroism. In the rock, muscovite is sporadically observed, as well as sericite and clay minerals formed as a result of feldspar weathering.

In granite, no porosity was observed during microscopic examination. According to the literature data [62], rocks of this type may have pores formed due to cracking, but practically no cracks were observed in the analyzed granite.

**Figure 13:**
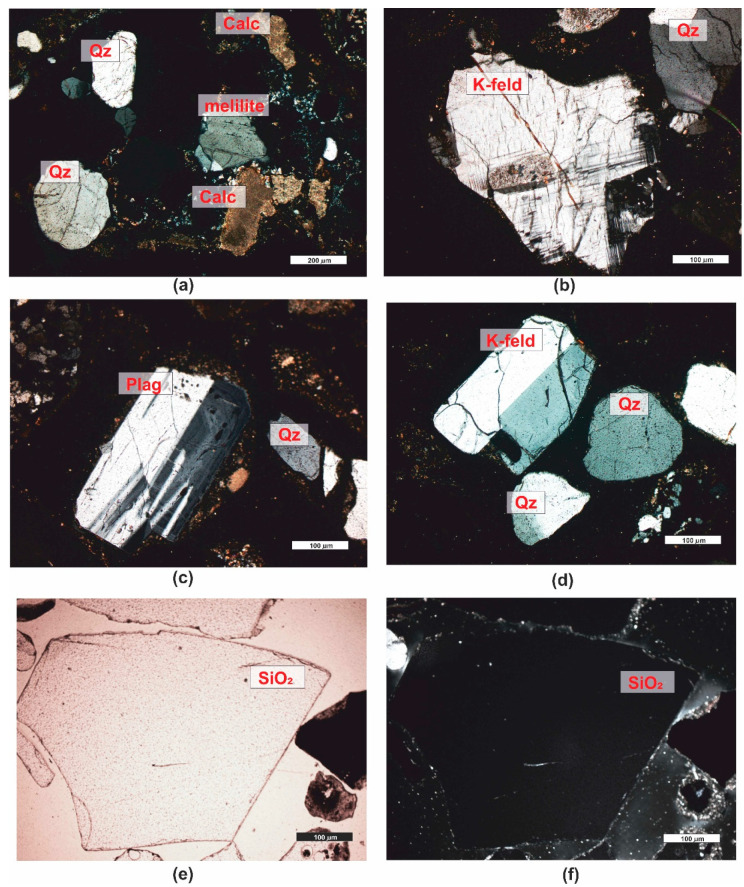
Microscopic photographs of waste slag PRO10. (**a**–**f**)—transparent light, thin section, XPL; (**e**) reflected light, thin section, PPL. Legend: see Figure 4.

*Waste slag (PRO10).* Slags and ashes resulting from the thermal incineration of municipal waste are characterized by a gray color, and unburned fragments of glass, metals, or ceramics can be seen with the naked eye. Macroscopically, this material somewhat resembles crushed or irregular fragments of sedimentary rocks (Figure 1). The resemblance to rocks becomes even more apparent during microscopic analysis. It has been observed that the waste, in terms of its structural–textural characteristics, shows similarities to clastic sedimentary rocks. The waste exhibits a psammitic-psefitic, different-grained structure. The grains composing the waste are usually well-rounded, although angular fragments are also present. The texture of the material, visible in larger fragments, is random and porous, sometimes even vesicular. The material contains a significant admixture of mineral substances described in the works of [44,63]. The primary mineral component of municipal slag is quartz grains. They occur in the form of oval, rounded, slightly fractured grains (Figure 13a–d). Due to the action of high temperature, this mineral exhibits undulatory extinction. Another mineral present is melilite. Melilite belongs to the group of group silicates Ca_2_Mg and Ca_2_Al. These minerals form at high temperatures (Figure 13a). They are characterized by low, first-order, interference colors and, as quartz, undulatory extinction. The surface of the grains is slightly fractured, with poorly marked cleavage. The mineral typically has an irregular or slightly oval shape. Another commonly occurring mineral in the waste is calcite, constituting an integral component of almost all samples. It occurs in the form of small sparitic and microsparitic clusters, and even sparitic aggregates (Figure 13a,c) with high, third-order, interference colors. The group of feldspar minerals is another set of minerals contributing to the composition of the incinerated waste. In the analyzed samples of slag, numerous grains of potassium feldspar and plagioclase are encountered. All feldspars have gray, low, first-order, interference colors, sometimes showing cleavage, and they exhibit multiple or singly twinned crystals. Mineral grains are often subhedral and tabular. The material also includes, among others, multiple twinned plagioclases (Figure 13c) and potassium feldspars (Figure 13b,d).

Additionally, in the slag, other minerals were present, including, among others, apatite, anhydrite or gypsum, and wollastonite.

As for the amorphous phase, each sample contains a significant admixture of materials of anthropogenic origin that have not undergone complete combustion in the incineration process. These primarily include glass fragments, which were observed even with the naked eye. Glass fragments or glaze, when observed under polarized light, are optically isotropic (completely extinguish light) (Figure 13e,f). The slag also contains a considerable amount of metallic phase, composed of shiny fragments of various, unseparated metallic substances in the recovery process [44]. These substances, in microscopic examinations under transmitted light, are opaque and isotropic.

**Figure 14:**
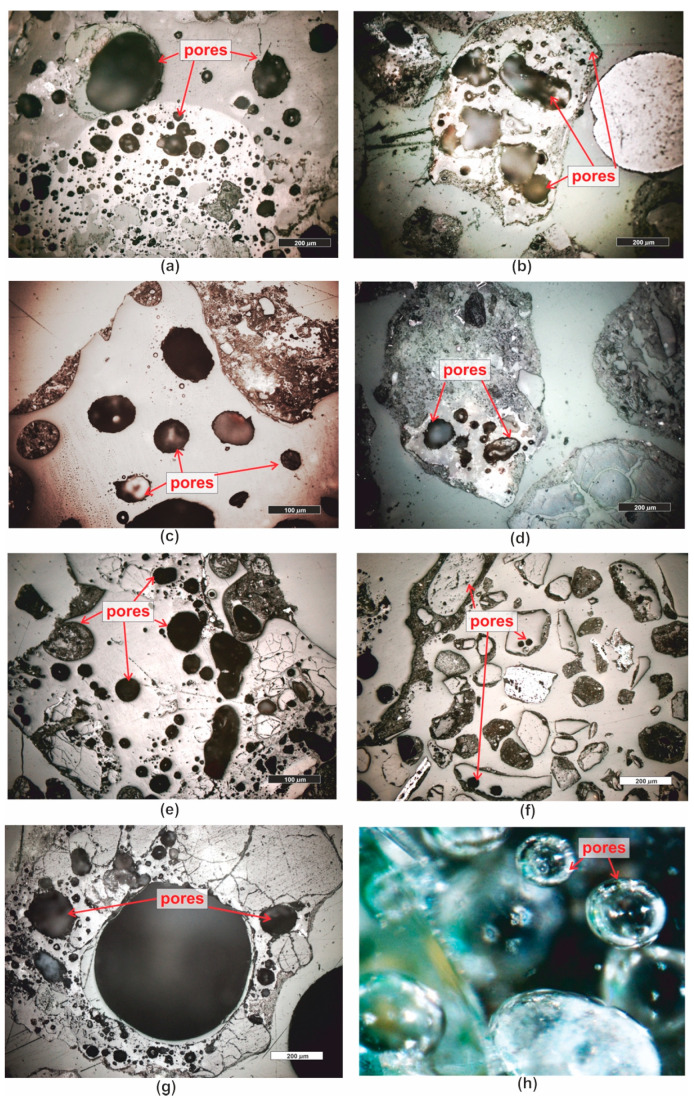
Microscope photographs of pores present in waste. (**a**–**g**) reflected light, polished section, PPL; (**h**) stereoscopic microscope, mag. 100×. Legend: see Figure 4.

Analyses of the pore space in the slag have shown numerous pores, typically oval, sometimes circular, and closed. Their size varies, ranging from the smallest, at the limit of microscopic visibility, to very large ones, even several millimeters in diameter (Figure 14a–h). Microscopic point analyses revealed that the porosity of the entire material in the samples varies from 9.07 through 12.92 up to 19.94%, depending on the analyzed grain size class, as reported in the study by [44] (Table 1). In the grain size class of 0.16–0.25 mm, which was analyzed in detail with other research methods, the porosity was 4.15%, while the largest and most numerous pores were present in the coarsest classes (Table 2).

### 3.2. Analysis of Low-Pressure Isotherms

**Figure 15:**
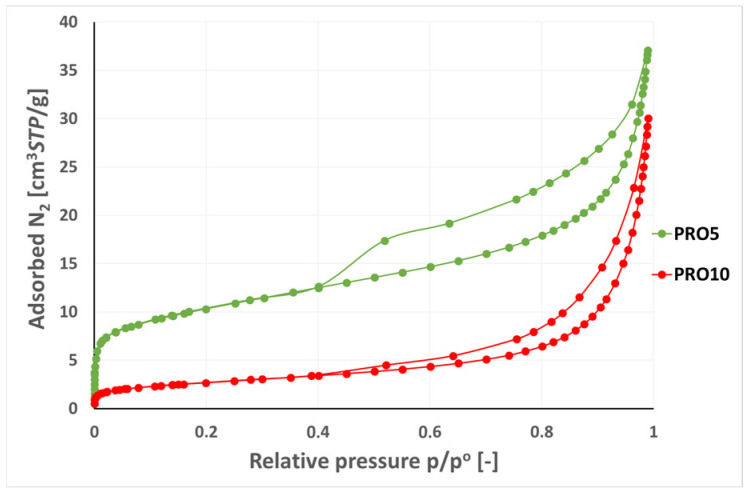
Nitrogen adsorption/desorption isotherms on the PRO5 and PRO10 samples, −196.0 °C.

**Figure 16:**
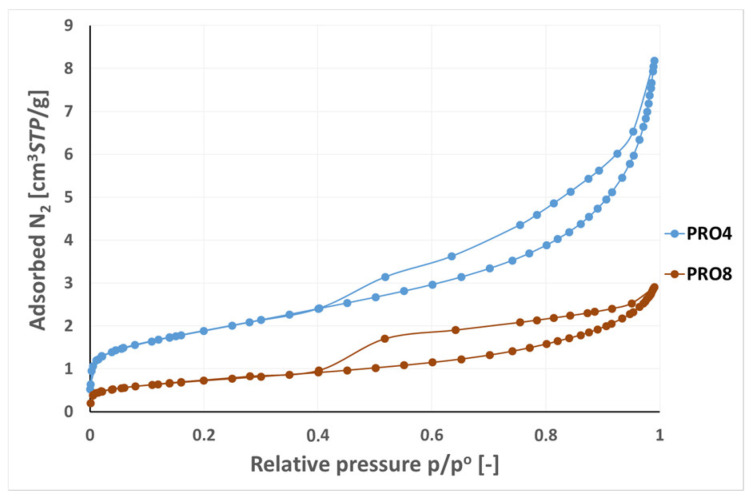
Nitrogen adsorption/desorption isotherms on the PRO4 and PRO8 samples, −196.0 °C.

**Figure 17:**
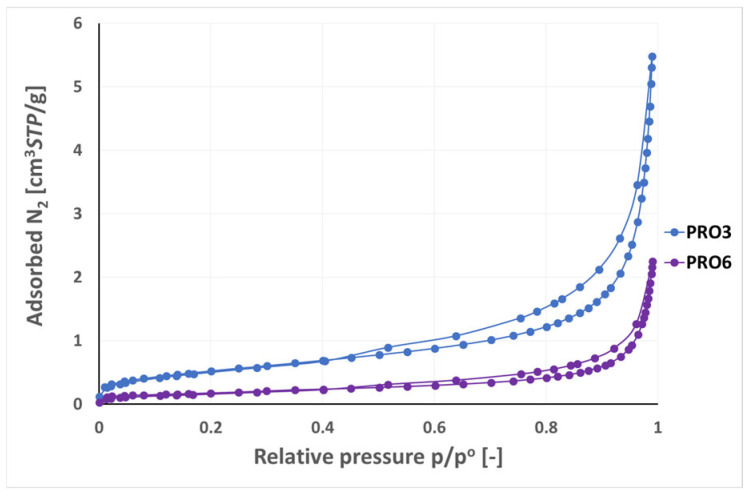
Nitrogen adsorption/desorption isotherms on the PRO3 and PRO6 samples, −196 °C.

**Figure 18:**
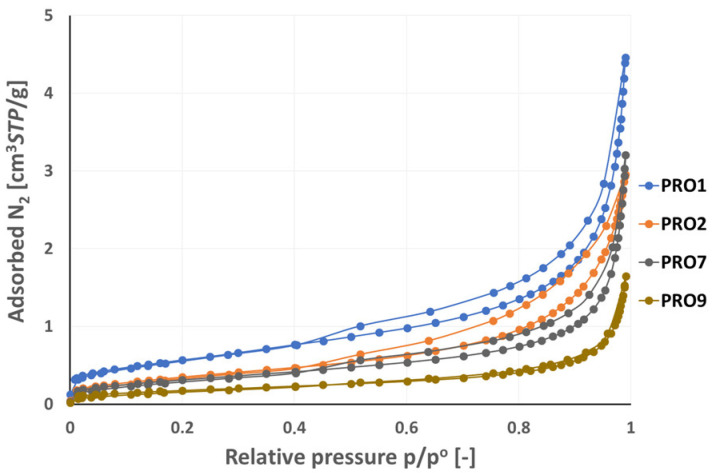
Nitrogen adsorption/desorption isotherms on the PRO1, PRO2, PRO7, and PRO6 samples, −196 °C.

The results of the low-pressure N2 adsorption studies are presented in Figure 15, Figure 16, Figure 17 and Figure 18 in the form of adsorption–desorption isotherm curves in the relative pressure range p/p° from 0 to 0.99. The isotherms are grouped on the individual figures due to similarities in the amount of adsorbed gas, course of the isotherm, and possible hysteresis.

Figure 15 shows the nitrogen adsorption–desorption isotherms for the PRO5 and PRO10 samples, which were characterized by the highest adsorption capacity among the tested materials, 37.06 cm^3^/g and 30.04 cm^3^/g, respectively. The obtained isotherms corresponded to type IV according to the International Union of Pure and Applied Chemistry IUPAC classification for physical adsorption [33]. Characteristic for type IV was the occurrence of a hysteresis loop and adsorption reaching the limit value at the high relative pressures p/p°.

For the PRO5 sample, the shape of the isotherm in the range of low relative pressures up to 0.02 was characterized by a strong increase, which was caused by the participation of micropores with dimensions < 2 nm. An increase in the range of low relative pressures was also observed for the PRO10 sample, but the adsorption capacity of this material was five times lower than that of the PRO5 sample (see Figure 15). In the p/p° range from 0.05 to 0.5, the isotherm of the PRO5 sample systematically increased, which indicated the formation of a multilayer of adsorbing gas molecules. This was characteristic for the gradual filling of transition pores of dimensions from 2 nm to 50 nm [33]. From the pressure value p/p° of around 0.8, the adsorption isotherm increased more strongly to the value of 0.99 (see Figure 15). In the range of p/p° from 0.1 to 0.6, the isotherm for the PRO5 sample increased slightly, while from the value of p/p° = 0.8, a strong increase in adsorption capacity was observed and at p/p° = 0.99, it reached a value similar to that of the PRO5 (37.06 cm^3^/g).

For both samples, the N_2_ adsorption and adsorption curves formed hysteresis at the p/p° range from 0.4 to 0.99. The obtained course of the isotherm was related to the capillary condensation of gas in the area of mesopores and macropores of the tested materials. The shape of the presented H1 hysteresis loop for the PRO5 sample, characterized by an almost symmetric adsorption and desorption curve, was related to the presence of pores with cylindrical geometry. In the case of the PRO10 sample, H3 type hysteresis, characteristic of slotted pores, was observed.

The nitrogen adsorption/desorption isotherms on the PRO4 and PRO8 samples corresponded to type IV according to the IUPAC classification [33]. The adsorption capacity of the PRO8 sample (2.85 cm^3^/g) was 2–3 times lower than that of the PRO4 sample (8.18 cm^3^/g, see Figure 16). In the p/p° range from 0.4 to 0.99, hysteresis of the adsorption and desorption branches were observed. Nitrogen capillary condensation occurred for both samples. The shape of the hysteresis loop for the PRO4 sample was of the H1 type and resulted from the presence of cylindrical pores. In the case of the PRO8 sample, H2 hysteresis was observed, which was asymmetric and slightly triangular in shape. This type of hysteresis characterizes materials with bottle-shaped pores or pore systems connected in a network. The PRO8 basalt sample had clay minerals in its structure, resulting from the weathering of basalt minerals. The surface properties in the mesopores range (2–50 nm) shown in the adsorption analysis resulted from the presence of a clay admixture.

Figure 17 presents the nitrogen adsorption–desorption isotherms for the PRO3 sample (micrite limestone) and PRO6 sample (graphite). According to the data obtained from the isotherms, the adsorption capacity toward nitrogen was at −196 °C more than twice higher for the PRO3 sample (5.48 cm^3^/g) than for the PRO6 sample (2.25 cm^3^/g). The obtained isotherms represented type IV isotherms according to [33]. For both materials, hysteresis loops were observed in the p/p° range from 0.4 to 0.99. From the pressure p/p° around 0.90, a strong increase in the amount of adsorbed gas was seen. The shapes of the hysteresis loops for the samples from Figure 17 were of the H3 type, characterized by slotted pores.

Among the samples presented in Figure 18, the PRO1 sandstone sample had the highest adsorption capacity (4.45 cm^3^/g), followed by the PRO7 limestone (3.21 cm^3^/g) and the PRO2 sandstone (2.96 cm^3^/g), while the PRO9 granite had the lowest adsorption capacity (1.65 cm^3^/g) among all the studied samples. Adsorption–desorption isotherm plots in the p/p° range from 0 to 0.99 corresponded to type IV according to the IUPAC, except for the PRO9 granite sample showing a type II isotherm [33]. Type II characterizes non-porous or macroporous materials with a pore diameter > 50 nm, while type IV, as described earlier, refers to mesoporous materials with a pore diameter from 2 to 50 nm. Granite, which generally does not have micropores or mesopores, may contain voids classified as macropores. This range of porosity may come from fractures that the rock may undergo. The PRO1, PRO2, and PRO7 samples showed an H3 type hysteresis loop characteristic of slotted pores.

### 3.3. Textural Parameters

**Figure 19:**
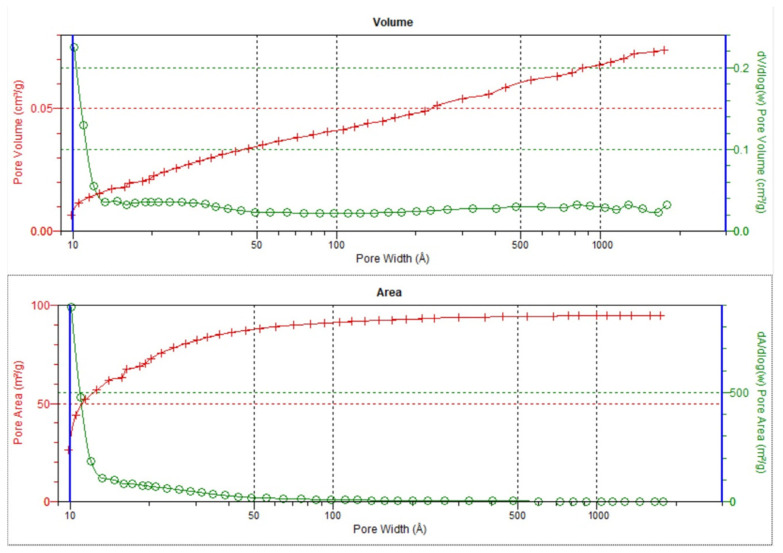
Sample PRO5: Pore volume and area distribution relative to pore diameter, BJH Adsorption, Harkins and Jura, Faass correction.

**Figure 20:**
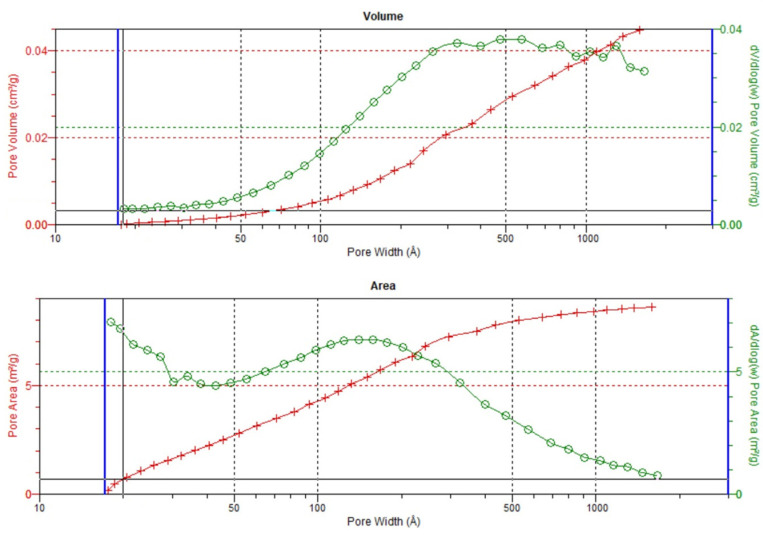
Sample PR10: Pore volume and area distribution relative to pore diameter, BJH Adsorption, Harkins and Jura, Faass correction.

Figure 19 and Figure 20 present the distributions of pore volume and area obtained from the analysis of N2 adsorption/desorption isotherms for the PRO5 and PRO10 samples. These samples stand out from other materials, which show significant similarity in pore distribution and weak surface features. Table 2 compares the textural parameters of all the tested materials.

As can be seen from Figure 19, the claystone sample PRO5 had micropores with dimensions of about 1 nm, the volume of which was insignificant, but the surface area was noteworthy in the amount of about a dozen m^2^/g of sample. The sample also contained fine pores from 2 to 5 nm, with a total volume of 0.015 cm^3^/g and a surface area of 20 m^2^/g. The PRO5 sample was microporous and mesoporous with microporosity responsible for the highest specific surface S*_BET_* among the tested materials. Additionally, for the PRO5 sample, the claystone had the largest specific surface area of which approximately 33% was the surface of micropores, while the remaining part was covered by the external surface, which consisted mainly of mesopores. Similarly, the determined micropore volume was 0.0205 cm^3^/g with a total pore volume of 0.0750 cm^3^/g (see Table 2).

As can be seen from Figure 20, the post-process slag sample PRO10 had a certain share of micropores with dimensions of approximately 2 nm, the volume of which was insignificant, but the surface area was noticeable in the amount of several m^2^/g of sample. The sample contained pores in the mesoporosity range from 2 to 50 nm, whose share in porosity was dominant at a volume of 0.03 cm^3^/g and a surface area of 8 m^2^/g. This sample, mesoporous with some microporosity, was responsible for the second highest value of specific surface area among the tested materials, amounting to approximately 9.5 m^2^/g. As can be read from Table 2, which lists the textural parameters of all the materials, for the post-process slag sample PRO10, the micropores area accounted for approximately 7% of the total specific surface area, while the remaining part of the specific surface area consisted of mesopores along with the external surface.

The remaining tested materials, according to Table 2, indicate meso- and macroporosity. The specific surface areas of the remaining samples did not exceed 2.5 m^2^/g, which translates into low adsorption capacities of the samples toward nitrogen (−196 °C). Micropores did not appear in the remaining samples. The samples had a certain number of mesopores ranging from 2 to 50 nm with a BJH average pore diameter of approximately 10 nm.

### 3.4. Densiometric Studies Results

Below, Table 3 presents the results of the pycnometric tests.

Table 4 summarizes the results obtained from the porosity measurements using various research methods. The results of adsorption and densimetric tests were compared with microscopic evaluation. As can be seen from Table 4, adsorption methods performed very well for the evaluation of micropores. The exception was black adsorbent with a microporous internal structure which is inaccessible for adsorption at low temperatures, e.g., −196 °C (see the microporous sample of PRO6—graphite, which has a low adsorption capacity in the low-pressure N2 adsorption test and specific surface). Pycnometric methods, on the other hand, measured porosity up to a certain maximum diameter of the void/gap; therefore, some part of the macropores might not be captured after immersion in the quasi-liquid that will fill the voids/gaps and they will not be taken into account in the test. It has been shown that microscopic methods successfully visualize the macroporous part of porosity, both macropores, voids, and internal cracks. They may also classify some of the transition pores as macropores. As a result, the methods for analyzing the porous structure of materials of various origins presented in this work showed that they are important tools for examining porosity, they overlap with each other, and none of them can be the exclusive method for assessing a given porosity range. The methods are complementary to each other.

## 4. Discussion and Conclusive Remarks

Based on the conducted research, considering microscopic analyses as the foundation and complementing with pycnometric, textural, and adsorption analyses, it was observed that the analyzed waste generated in the incineration process at the waste-to-energy plant shared many similarities with rocks. It can be successfully described using petrographic–stereological methods. This waste exhibited many common features with clastic sedimentary rocks such as sandstone, conglomerate, or mudstone, and relatively few similarities with igneous rocks. Based on the research, similarities and differences between the waste and selected clastic rocks were determined and compiled in Table 5. Analyzing the waste using microscopic methods allows for the assessment of broadly defined structural–textural features, conducting a mineralogical characterization (assuming a mineral phase is present), and describing the pore space, the nature of pores (closed-open), etc. Stereological analyses also enable the precise determination of porosity parameters within the range of pores from about 1 μm to the size of the sample, which cannot exceed the size of a microscope stage (about 10 cm^2^), according to [33], in the range of macroporosity.

Optical microscopy has yet another crucial feature—it allows for the precise characterization and measurement of macropores, not only open but also closed, which poses a challenge for methods such as porosimetry. Microscopic analyses, although highly accurate for macroporous materials, are unable to determine the entire range of porosity in a rock characterized by meso- and/or micropores.

Detailed characterization of the texture of the tested samples, obtained on the basis of low-pressure nitrogen adsorption and desorption isotherms at −196 °C on the ASAP2020 apparatus, showed similarities and differences in the values of the obtained parameters of the porous structure depending on the type of sample. Pycnometric measurements showed that the real density and apparent density, used to determine the porosity of the samples, complemented the microscopic and low-pressure adsorption methods. All methods of analyzing the pore space of rocks complement each other and together constitute a complete set of methods for analyzing the pore structure.

The conducted research has shown that optical microscopy is a very important analysis, but only in combination with other methods (e.g., pycnometry) does it give a full picture of the nature and size of the pore space of the analyzed natural materials and slag and ash generated in municipal waste incineration plants.

The next stage of research concerning post-process waste, resulting from municipal waste incineration in a thermal waste treatment plant, will involve detailed mineralogical analyses based on X-ray diffraction and scanning electron microscopy analysis (SEM/EDS). This research, coupled with studies on the leaching of harmful substances (e.g., using the Tessier [47] or BCR method [64]), will help identify the specific mineral substances that might significantly impact the environment. Preliminary analyses suggest that the waste is relatively safe and does not pose a threat to the natural environment, but the results of these studies will be published in subsequent scientific articles.

## Figures and Tables

**Table 1:** Porosity of grain classes according to [44].

**Grain fraction**	4–2.5	2.5–1.6	1.6–1	1–0.63	0.63–0.4	0.4–0.25
**Porosity [%]**	19.81	9.97	11.55	11.32	5.87	4.15

**Table 2:** Textural parameters of the tested materials obtained from the analysis of low-pressure nitrogen adsorption isotherms.

		N_2_, −196 °C										
		PRO1	PRO2	PRO3	PRO4	PRO5	PRO6	PRO6	PRO7	PRO8	PRO9	PRO10
**Adsorption capacity (a_N2_)**	**cm^3^/g**	4.44	2.95	5.48	8.18	37.06	2.96	2.245	3.21	2.908	1.6498	30.04
**BET surface area** **0.05 < p/p° ≤ 0.35**	**m^2^/g**	2.080	1.320	1.920	6.650	35.800	1.670	0.642	1.161	2.560	0.650	9.490
**t-plot micropore area**		0.000	0.000	0.v000	0.515	12.070	0.000	0.249	0.000	0.101	0.000	0.694
**t-plot external surface area**		2.080	1.290	1.922	6.133	23.014	1.654	0.476	1.170	2.449	0.619	8.791
**BJH cumulative surface area of pores 1.7–300 nm**		2.080	1.320	1.996	5.900	35.800	1.670	0.630	1.237	2.550	0.670	8.700
**BJH total pore volume (V_t_)**	**cm^3^/g**	0.0069	0.0046	0.0085	0.0127	0.0750	0.0046	0.0035	0.0048	0.0045	0.0026	0.0465
**t-plot micropore volume**		0	0	0	0.0003	0.0205	0	0.0001	0	0.0001	0	0.0032
**BJH cumulative volume of pores 1.7–300 nm**		0.0068	0.0045	0.0082	0.0129	0.0545	0.0044	0.0034	0.0047	0.0046	0.0024	0.0446
**BJH average pore diameter**	**nm**	12	12	16.3	7.39	7.05	9.2	19.2	15.1	6.7	13.6	18

**Table 3:** Textural parameters of the tested materials obtained from the analysis of low-pressure nitrogen adsorption.

Sample	Real Density [g/cm^3^]	Aparent Density [g/cm^3^]	Porosity [%]	Pore Volume [cm^3^/g]
**PRO1**	2.6926	2.3592	12.4	0.0525
**PRO2**	2.6707	2.3000	13.9	0.0603
**PRO3**	2.7247	2.4384	10.5	0.0431
**PRO4**	2.7108	2.4906	8.1	0.0326
**PRO5**	2.5252	2.2260	11.8	0.0532
**PRO6**	1.8681	1.4303	23.4	0.1639
**PRO7**	2.7073	1.8554	31.5	0.1696
**PRO8**	2.7550	2.6453	4.0	0.0151
**PRO9**	2.7895	2.5907	7.1	0.0275
**PRO10**	2.5850	1.9570	24.3	0.1241

**Table 4:** Summary of the porosity test results using various measurement methods.

Sample	Microscopic [% Porosity]	Densimetric [% Porosity]	Adsorption [% Porostiy]
	>1000 nm (Macroporosity)	<25,000 nm (Micro, Meso, Macro)	<300 nm (Micro and Meso)
**PRO1**	17.5	12.4	0
**PRO2**	11.9	13.9	0
**PRO3**	11.6	10.5	0
**PRO4**	10.4	8.1	2.7
**PRO5**	0	11.8	8.5
**PRO6**	25	23.4	2.6
**PRO7**	26.4	31.5	0
**PRO8**	1.5	4.0	2.0
**PRO9**	0	7.1	0
**PRO10**	4.2	24.3	6.9

**Table 5:** Comparison of the possibility of describing selected features and microscopic analyses on clastic sedimentary rock and on waste generated after municipal waste incineration.

**Comparison—Features/Analysis**	**Clastic Rock of the Sandstone Type**	**Slags and Process Ashes**
**Color/pleochroism**	Yes	Yes/maybe
**Relief**	Yes	Yes
**Cracks and pores**	Yes	Yes
**Structure and texture**	Yes	Yes or not
**Cement, matrix**	Yes	If present, yes
**Shape and size of ingredients**	Yes	Yes
**Interference colors**	Yes	Yes or not
**Crystal twinning**	Yes	Yes or not
**Qualitative assessment**	Yes	Yes or not
**Stereological analyses—quantitative analysis**	Yes	Yes

## Data Availability

The data presented in this study are available on request from the corresponding author.
